# US Medical Schools’ 2024 Commencements and Antisemitism: Addressing Unprofessional Behavior

**DOI:** 10.5041/RMMJ.10537

**Published:** 2025-01-30

**Authors:** Steven Roth, Hedy S. Wald

**Affiliations:** 1 Michael Reese Professor of Anesthesiology and Vice Head for Research and Faculty Affairs, Department of Anesthesiology, University of Illinois College of Medicine; Professor Emeritus, University of Chicago, Chicago, Illinois, USA; 2 Clinical Professor of Family Medicine, Warren Alpert Medical School of Brown University, Department of Family Medicine, Providence, Rhode Island, USA

**Keywords:** Activism, antisemitism, commencement, humanities, professionalism, regalia

## Abstract

**Introduction:**

Antisemitism and antisemitic incidents have been increasing in United States medical institutions since the Hamas attack of October 7, 2023. Such incidents include anecdotal reports of antisemitic displays at medical school commencements. This study examined unprofessional behavior observed at the commencement ceremonies of the 25 US medical schools top-ranked for research excellence. This issue is significant since these graduates are expected to become future leaders in the field of medicine.

**Materials and Methods:**

Based on publicly available videotaped commencements, we assessed the number of students in the graduating classes wearing non-school-provided regalia, carrying signs, wearing protest buttons, or engaging in verbal protests related to the Israel–terror groups conflict that were either openly antisemitic or potentially offensive or insensitive.

**Results:**

Symbols representing antisemitic themes (keffiyehs and three-part graduation stoles conveying antisemitic messages) were worn by students at just over half (13) of the medical schools. The mean number of students in each school wearing keffiyehs or non-official school stoles was 4.0 (95% confidence interval [CI] 2.2–5.8), ranging from 0%–13% of the classes, or 2.5% of the overall graduating cohort. The wearing of buttons, carrying of banners or signs, verbal protests interrupting the ceremony, or students deviating from script ranged from 0% to 22.5% of graduating students, with a mean of 2.7 per school (95% CI −0.8–6.2), or 1.7% of the medical schools graduating cohort.

**Conclusions:**

We identified unprofessional behavior at commencements of top-ranked medical schools consisting of antisemitism and displaying offensive and insensitive symbols and messaging. There is an urgent need for medical schools in the US to educate medical trainees about the dangers of antisemitism and all forms of hate and insensitivity.

## INTRODUCTION

Advocacy to improve health outcomes is a physician’s professional responsibility. Its incorporation as a competency into medical curricula has been encouraged^1^ and further elaborated within calls for “medical activism” to include “moral determinants of health.”^2^ Advocacy and activism are rooted in the ability to be socially engaged while exhibiting clear and responsible communication with patients and the public, fundamental skills which we, as responsible medical educators, regularly role-model for, or teach, our medical students to emulate.^3^ In line with these competencies, academic medical organizations have issued and continue to produce statements on topics impacting health, including pandemic management, firearms, climate change, international conflicts, and social justice.

Unprofessional behavior among medical trainees, which includes communication issues and can relate to advocacy or activism, is, however, common.^4^,^5^ It has been reported that a majority of trainees are unable to accept responsibility for their unprofessional behavior.^6^ This is of concern given that disciplinary action by medical licensing boards against physicians is highly associated with prior unprofessional behavior in medical schools.^4^ Unprofessional communication is reportedly the most common source of co-worker complaint reports against physicians.^7^

A recent increase in a particular form of unprofessional behavior among medical students is of concern, receiving attention within medical education and the media. Students have made hateful or offensive statements on social media and directly to fellow students, including antisemitic content, related to the conflict between Israel and terror organizations that began with the deadly Hamas invasion of Israel on October 7, 2023.^8^–^10^ Several disturbing incidents have also been reported at United States (US) medical schools. These include the tearing down of posters displaying Jewish hostages, including children and US citizens held by Hamas, which is recognized by the US and most Western countries as a terrorist organization.^11^ Additionally, there have been accusations that Jewish students are complicit with genocide, instances of Holocaust distortion or inversion, and the inclusion of antisemitic content in medical school coursework.^8^,^12^

As medical school educators, we noted certain behaviors at the commencement ceremonies of our schools by reviewing publicly available video recordings. Students were observed wearing symbols associated with the conflict between Israel and terror organizations, as well as making public verbal protests. Some of the symbols and protests conveyed antisemitic themes, while others could be interpreted as offensive, insensitive, or harassing and violating standards of responsible professionalism. Despite having been instructed to wear only school-provided regalia, some students wore non-professional items, further breaching the tenets of professionalism. This raised the question: was this behavior common at other medical schools or just a local phenomenon?

Commencements, amongst the most hallowed of academic celebrations, are traditionally seen as solemn occasions. The symbols and clothing people choose to wear reflect their thinking, attitudes, and worldviews. Unfortunately, depending upon their content, these expressions can deviate from positive intentions, instead conveying hatred, insensitivity, or offensiveness. This is the antithesis to creating “knowledgeable and *sensitive* health care providers” as espoused in the medical humanities framework^13^ and the aims of diversity, equity, and inclusion.^14^

This study examined student behavior for displays of antisemitism via behavior or regalia at the commencements of top-rated US medical schools. These actions are significant because stakeholders expect graduates of these medical schools to become future leaders in medicine. Furthermore, other medical schools worldwide typically look to them for leadership and innovation. Hateful, offensive, or insensitive behavior, particularly at recorded official events available to the public, can damage public trust in medicine, potentially leading to negative publicity, donor attrition, and a further erosion of trust.^15^

## METHODS

### Data Availability and Privacy Concerns

The study was deemed “exempt” by the Institutional Review Board of the University of Illinois at Chicago. We studied the commencement ceremonies at the 25 US medical schools top-ranked for research excellence in the *United States News and World Report* for 2023–24.^16^ The 2024–25 rankings were unavailable at the time of the commencements and the start of this study. Graduating class sizes were obtained from the Association of American Medical Colleges (Washington, DC) FACTS database.^17^ All commencements were professionally videotaped by the schools and publicly available on each medical school’s website, mostly as YouTube videos. Permissibility to view and report on publicly available videos of students is partly governed by the US Family Educational Rights and Privacy Act (FERPA).^18^ This law gives parents certain rights over their children’s education records. Under FERPA, a video of a student is considered an education record if it is directly related to the student and is maintained by an educational institution or a party acting on its behalf.^19^ Commencement videos are not considered by FERPA to be directly related to a student,^20^ hence the images may be freely viewed and published.

### Definition of Antisemitism

For the purposes of this research, antisemitism was defined according to the definitions, in part, of the International Holocaust Remembrance Alliance (IHRA) antisemitism guidelines,^21^ adopted by the US National Strategy to Counter Antisemitism,^22^ the US State Department,^23^ the 35 IHRA member countries, including the USA and much of the European Union,^22^,^24^ and the Global Imams Council, consisting of over 1,500 imams from 80 different countries.^25^ In brief, all behavior and/or regalia, including symbols, calling for aiding or justifying the killing or harming of Jews in the name of a radical ideology or extremist view of religion, or denying the Jewish people their right to self-determination, or declarations that the existence of the State of Israel is a racist endeavor (i.e. an apartheid state) were considered antisemitic.

### Data Analysis

Schools and their *US News and World Report* ranking (1–25) are listed in Table 1. Commencement videos were not available online for 7 schools (Weill-Cornell College of Medicine, Northwestern Feinberg School of Medicine, New York University Grossman School of Medicine, University of Chicago Pritzker School of Medicine, Washington University-St Louis School of Medicine, University of Texas Southwestern Medical School, and University of California at Los Angeles David Geffen School of Medicine); however, University of Chicago, Washington University, and University of Texas Southwestern provided full commencement videos upon request. Of the remaining four, two schools advised that no full-length videos were available (Northwestern Feinberg, and NYU Grossman); however, highlight videos were available on two of the school commencement websites and/or on social media (UCLA David Geffen, and Weill-Cornell). A California Freedom of Information Act request was filed with UCLA in June 2024; to date, the full commencement video for the School of Medicine has not been provided.

**Table 1:** Students Wearing Antisemitic or Offensive Stoles or Keffiyehs, and Those Wearing Protest Buttons, Carrying Signs, or Protesting Verbally or with Banners.

Medical School Rank*	Medical School Name	Class Size	Stoles or Keffiyehs (%)	Signs, Buttons, and Protests (%)
1	Harvard University	182	10 (5.5%)	13.0 (7.1%)

2	Johns Hopkins University	112	1 (0.9%)	0 (0%)

3	University of Pennsylvania Perelman	135	0 (0%)	0 (0%)

4	Columbia University Vagelos	142	8 (5.6%)	1.0 (0.7%)

5	Washington University in St Louis	67	0 (0%)	0 (0%)

5	Stanford University	76	11 (14.5%)	3.0 (3.9%)

5	University of California San Francisco	173	10 (5.8%)	39.0 (22.5%)

5	Duke University	133	0 (0%)	0 (0%)

5	Vanderbilt University	95	0 (0%)	0 (0%)

10	Cornell University (Weill)	109	Not available	3.0 (2.8%)

10	Yale University	103	8 (7.8%)	0 (0%)

13	University of Pittsburgh	126	0 (0%)	0 (0%)

13	University of Michigan	181	9 (5%)	0 (0%)

13	Northwestern University (Feinberg)	141	Not available	Not available

13	New York University (NYU) Grossman	110	Not available	Not available

13	University of Washington (UW Medicine)	254	0 (0%)	0 (0%)

13	Mayo Clinic (Alix)	104	1 (1.0%)	0 (0%)

18	Icahn School of Medicine at Mount Sinai	133	4 (3.0%)	0 (0%)

18	University of Chicago (Pritzker)	92	0 (0%)	0 (0%)

18	University of California, Los Angeles David Geffen	168	7 (4.2%)	Not available

21	University of California San Diego	144	4 (2.8)	0 (0%)

22	Baylor	170	10 (5.9%)	0 (0%)

23	Emory University	134	5 (3.7%)	0 (0%)

24	U Texas Southwestern	228	0 (0%)	0 (0%)

25	Case Western Reserve University	210	0 (0%)	0 (0%)

	Mean (SD)	140.9 (45.3)	4.0 (4.2)	2.7 (8.4)
	CI	123.2–158.5	2.2–5.8	−0.8–6.2

	Totals for all students (%)	3,522	88 (2.5%)	59 (1.7%)

* School rankings are based on the *US News and World Report*.^16^ Ties in the rankings are due to US News and World Report ranking methodology.

Data in the two rightmost columns are provided as numbers and % of those in each graduating class, and mean, standard deviation (SD), and 95% confidence intervals (CI). A total of 3,522 students were in the graduating classes. Where complete videos were not obtained, data are shown as “not available,” although partial results were obtained from highlights for Cornell and UCLA.

Microsoft Excel (Microsoft® Excel® for Microsoft 365 Microsoft Office, Version 2410 Build 16.0.18129.20158, 64-bit, Microsoft Corporation, Redmond, Virginia, USA) was used to record and calculate all data. We calculated the absolute number, percentage, and mean number of students in graduating medical school classes who wore regalia (beyond the officially provided gowns, caps, and stoles), carried signs, wore protest buttons, or participated in verbal protests related to the Israel–terror groups conflict. We noted if those actions were antisemitic or potentially offensive to others. We also recorded any non-official regalia, including stoles, buttons, or signs with the images or flags of countries other than the United States, or unrelated signs or messages. After setting specific review criteria, we watched all the videos and compiled the results in an Excel spreadsheet. Blinding to school identities was not possible since each video included banners and audio identification of each school.

Screenshots were taken from the videos using Google YouTube Screen Capture to generate images for publication. All data and images were stored on University of Illinois BOX, which is both FERPA- and HIPAA (Health Insurance Portability and Accountability Act)-compliant. Data were presented as mean and 95% confidence intervals. Images presented were either cropped or blurred to anonymize student identities.

## RESULTS

**Figure 1:**
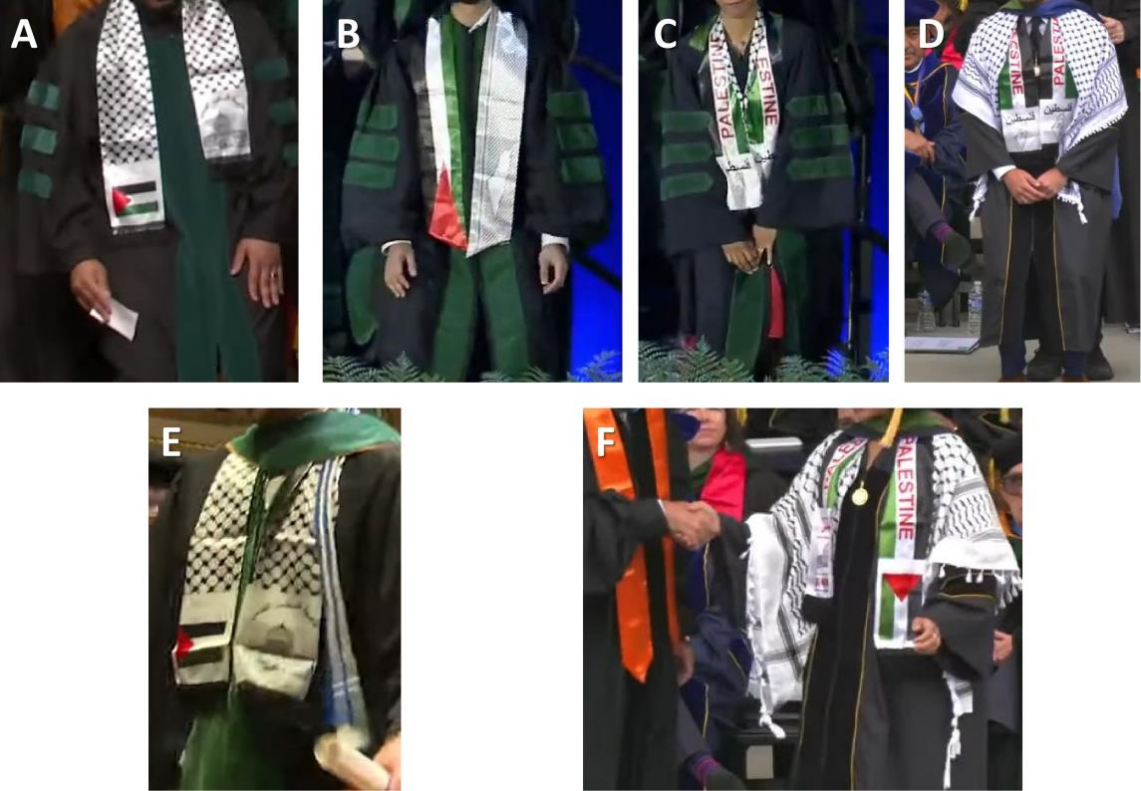
Examples of Antisemitic Regalia Worn by Medical Students at Three Medical Schools (Michigan, Baylor, and UCSD) During Commencement Ceremonies. **Panel A** presents the three-part stole consisting of a keffiyeh, the Palestinian flag, and the Dome of the Rock with an Arabic phrase, translated as “Jerusalem is Ours,” as worn by a student. Panels B through F show variations including the empty map of Israel and adjacent Arabic writing meaning “Palestine.” All photos were taken directly from the publicly available medical school commencement videos on YouTube.com using the Google Screenshot app.

The mean graduating class size was 141 (95% confidence interval [CI] 123–159), with a total of 3,522 graduating students across the 25 medical schools. Symbols suggestive of support for terrorism, or hateful antisemitic regalia, were worn by students at just over half (13) of the schools (Table 1). These included keffiyehs, which were worn over or under graduation gowns and still visible around the neck, and graduation stoles with antisemitic messaging. While keffiyehs may have cultural roots, today they are a symbol associated with political violence and support of terrorism; they are often worn by leaders of terrorist organizations and financial or material supporters of terrorism who call for the murder of Jewish people.^26^,^27^ The mean number of students wearing keffiyehs or such stoles in each school was 4.0 (95% CI 2.2–5.8), ranging from 0% to 13% of the classes, and constituted 2.5% of the overall graduating student cohort. In 6 schools (University of California at San Francisco (UCSF), Baylor School of Medicine, University of Michigan School of Medicine, Harvard Medical School, Weill Cornell, University of California at San Diego (UCSD) School of Medicine), students wore various three-part stoles consisting of a Palestinian flag, keffiyeh, and an empty map of Israel, some with the word “Palestine” adjacent to the map, written in Arabic. Other keffiyeh stoles contained a picture of the Dome of the Rock located over the site of the holiest place in Judaism, the Holy Temple, and “Jerusalem is Ours” adjacent, also written in Arabic (Figure 1). Such stoles symbolize support for the US Department of State-recognized terrorist organizations, Hamas, Palestinian Islamic Jihad, and Hezbollah.^11^ They imply the desire for annihilation of all Jews and the destruction of Israel, as represented by the empty map and the phrase “Jerusalem is Ours,” which suggests a desire for no Jewish presence in the city.^28^

Students at the authors’ institutions also wore keffiyehs and/or these three-part stoles, which met the definitions of antisemitism.

**Figure 2:**
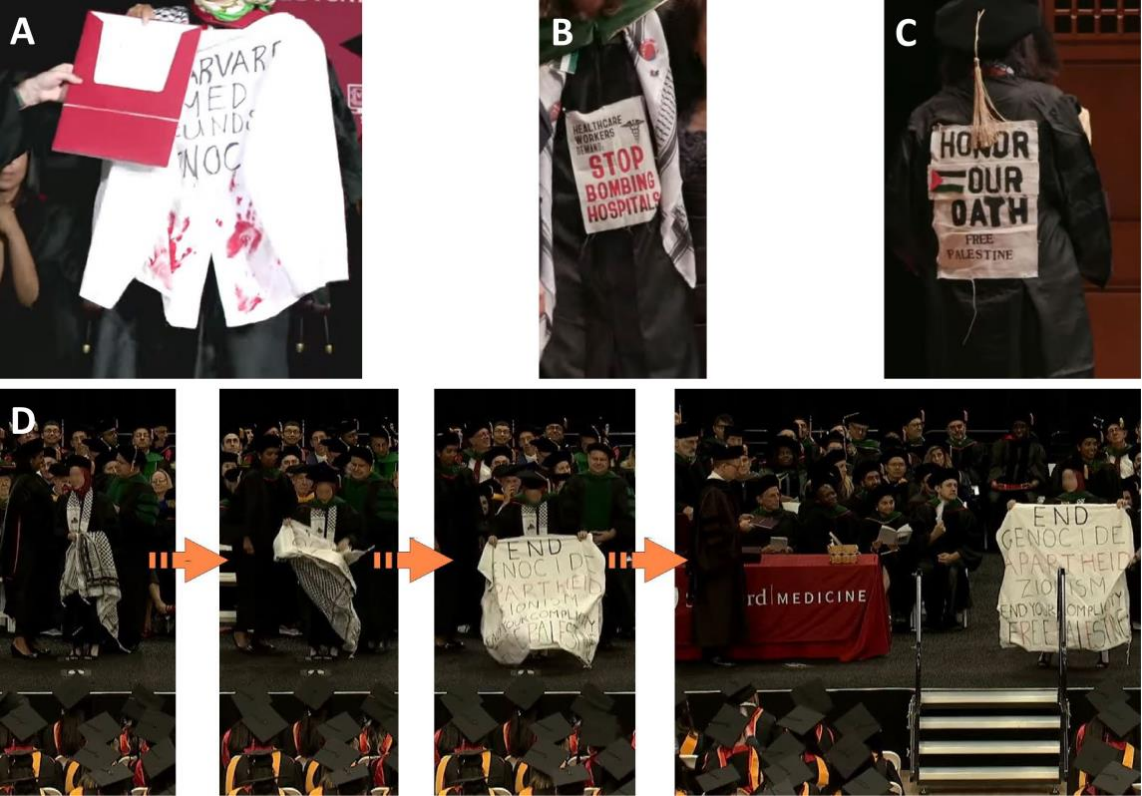
Examples of Antisemitic Signage Carried by Medical Students at Commencement Ceremonies of Another Set of Medical Schools (Harvard, UCSF, and Stanford). **Panel A** shows a Harvard student receiving her diploma while holding up to the audience a short white student coat with red-stained palm prints on the coat reading “Harvard Med funds genocide.” **Panels B** and **C** present two different signs worn by medical students over their gowns at UCSF. **Panel D** presents a student (blurred face) carrying a large keffiyeh in violation of Stanford School of Medicine policy (no carrying of any items permitted onto the stage), unfurling it, and revealing a sign on its reverse (orange arrows). The student waved the sign from side to side for about 30 seconds, taking care to face the Dean of the medical school (far left, last panel on the right). The sign reads, “End genocide, apartheid, Zionism, end your complicity, free Palestine.”

Additional antisemitic symbols, or, at the least, inflammatory, hateful, offensive, and/or insensitive messaging, were worn or carried by students. Banners, buttons, and signs were worn or carried that called for “divest now” at four schools (Harvard, Columbia University-Vagelos College of Physicians and Surgeons, UCSF, Stanford). “Divest now” refers to the boycott, divest, and sanction (BDS) movement aimed at Israeli academia and businesses (including US firms doing business with Israel);^29^ many founding goals of the BDS movement meet the definition of antisemitism, also known as the three “*Ds*” of antisemitism: demonization, delegitimization, and double standards.^30^–^33^ Other signs the students carried or wore on their gowns or caps included “occupation is a health crisis,” “stop bombing hospitals,” “Harvard Med funds genocide,” and “end genocide, apartheid, Zionism, end your complicity, free Palestine,” among others (Figure 2, Panels A, B, C). Harvard and UCSF, respectively, had particularly high percentages of students displaying anti-Israel protest banners supporting BDS (7%) or wearing buttons calling for BDS (22.5%). Students also delivered verbal protests, in most instances deviating from the ceremony programs.

The following examples illustrate the misuse of the term “genocide,” a legally determined definition with special status in international law.^34^,^35^

At UCSF, a graduating medical student had been asked to read a line of the Physician’s Oath in Arabic, for the announced purpose of “showcasing the rich diversity of the graduating class”; fluent speakers of foreign languages were thus asked to read one line of the oath. However, the student gave an impromptu speech accusing the State of Israel of “genocide.” There was no reader of Hebrew, but it was unclear if this was because of the absence of a native speaker or another reason.

At Baylor, a medical student chosen to speak on behalf of the medical school graduating class focused solely on the war in Gaza. The student accused Israel of “genocide,” and claimed that the medical school faculty had “actively stopped our attempts to stop genocide.”

At Weill-Cornell, two graduating medical students delivered on-stage protests against “genocide” in Gaza. Both students addressed the faculty loudly and disrespectfully. One grabbed their diploma and shouted “Free Palestine,” while the other student displayed a large sign reading “stop genocide,” which obscured their body.

Also at Weill-Cornell, medical students marched around the perimeter of the audience shouting “Free, Free Palestine.” This proclamation is often associated with unfounded and derogatory anti-Israel accusations, including antisemitic calls to replace the State of Israel and denial of the historical and religious connection of the Jewish people to the land of Israel.^36^

Outside the graduation ceremony at Warren Alpert Medical School of Brown University, medical students from years 1–3 shouted “From the River to the Sea,” a euphemism included in the Hamas terrorist charter conveying the desire for annihilation of the Jewish people and the State of Israel.^37^

At Stanford, a medical student wearing a keffiyeh also carried another large keffiyeh in her arms to the stage, in violation of school rules. When she unfurled the second keffiyeh, an antisemitic sign was revealed on its reverse side (Figure 2D).

Large signs protesting “genocide,” “faculty complicity,” as well as calling to “end apartheid” and “end Zionism” were carried onto the stage and displayed by medical students at Harvard, UCSF, and Stanford (Figure 2 and Table 1). Antisemitic actions included signs or statements demonizing Jews, as well as Holocaust inversion, i.e. accusing Jews of genocide.^21^

Overall, from 0% to 22.5% of graduating medical students protested in the form of buttons, banners, signs, or verbal protests, with a mean of 2.7 per school (95% CI −0.8–6.2), or 1.7% of the entire graduating cohort.

We were unable to find rules on the commencement websites at most of the medical schools studied regarding permissible items at the ceremonies. However, at Stanford and the University of Illinois-Chicago College of Medicine, medical students violated the stated prohibitions against wearing non-official regalia or carrying signs into the commencement ceremony. This is a professionalism violation of failure to obey rules.

## DISCUSSION

The moral imperative to counter antisemitism in medicine in the US exists within a context of increased antisemitism since October 7, 2023 in US medical schools, which has impacted the learning environment.^8^ Although this study focused on US medical schools, a significant rise in antisemitism has also been reported in medical schools in Canada and the United Kingdom (UK) since October 7, 2023.^38^–^40^

Furthermore, no anti-Palestinian or anti-Muslim signs or symbols, nor buttons or signs apart from those described above were noted in any of the studied commencement ceremonies. The national flags of other nations were worn at only two medical schools, with a total of five students in the entire graduating cohort.

Common language in the commencement oaths includes: “I will not permit considerations of … creed, ethnic origin, … or any other factor to intervene between my duty and my patient.” Hence, the unprofessional displays of offensive regalia, insensitive symbols, or antisemitism by the graduating medical students represent a clear violation of the oath they took during the commencements. This raises the question: can physicians who display such unprofessional behavior at a public ceremony communicate with and care for all their patients in a humanistic and non-biased manner?

Commencement ceremonies are hardly the place for disruptive behaviors, displays, and disputes related to geopolitical conflicts and emotionally laden controversial claims that misrepresent the issues at hand.^41^,^42^ Besides expressing offensiveness and sentiments of hate for Jews, the wearing of non-approved inappropriate regalia compromises the sensitivity and safety of fellow students, family, faculty, and attendees. Hateful displays, insensitivity, disrespect, disruptions of ceremonies, support for terror, disobedience of school rules, and false claims are unprofessional behaviors occurring in the context of a hallowed and public ceremony.^43^ Medical school curricula have increasingly emphasized sensitivity to all patients and peers. Cultivating sensitivity within medical education and practice is essential, considering its critical role within the physician–patient relationship, which includes fostering public trust.^44^ It must be emphasized that certain symbols, imagery, or speech content can be deeply offensive or hurtful to individuals of specific ethnicities, religions, or other specific backgrounds.

The unprofessional and inappropriate actions and expressions of hate observed during the commencements present an important opportunity for providing educational guidance on professional behavior. Various factors may contribute to the lack of sensitivity and respect demonstrated in the above examples, including potential misunderstandings regarding upholding professionalism within what may be perceived as “advocacy” or “activism.” Hence the “4 *E*s” paradigm (Education, Engagement, Empathy, and Enforcement) for countering antisemitism may be helpful.^8^ Education includes learning about Jewish identity and antisemitism, eliminating bias, and the historical role of medicine during the Holocaust. Engagement includes understanding the role of respectful civil discourse in medical education. Empathy within medical practice and the learning environment, particularly for students and families at significant occasions, can have a ripple effect on physician–patient communication and care. Enforcement conveys that standards, such as regalia policies, must be upheld, and provision of a non-hostile learning environment must be maintained.^8^ With regard to this latter item, the wearing of official regalia is generally associated with a more dignified ceremony, embodying the professionalism expected of medical graduates. For future commencements, proactive action that permits only official school-provided regalia, and no signs, is recommended. The authors are aware of several medical schools that have already issued such directives (private communication November 2024, Dr Edward Halperin, Chancellor and Chief Executive Officer of NY Medical College, Valhalla, NY, USA).

Universities and medical schools are being challenged to maintain the free exchange of ideas, essential to the advancement of medical science and improved healthcare, while appropriately addressing the unprofessional behavior of students or faculty considered offensive, insensitive, or overtly hateful. Such unprofessional behavior could be viewed by the public as representative of all medical student behavior or training. In light of concerns about academic freedom and First Amendment rights, legal guidance should be sought regarding the enforcement of relevant regulations at medical school events. With respect to displays of hate and offensiveness, which carry potentially serious ethical and legal consequences, institutions, particularly private ones, would be justified to limit what is allowed to be worn or carried at commencement ceremonies.^45^ The ability to restrict such behavior may be more limited in a public university, given potential for First Amendment challenges.^46^ United States institutions of higher learning (including medical schools), however, are obligated to provide protections for all students within the Title VI Civil Rights Code, which prohibits the existence of a hostile learning environment on the basis of “shared ancestry” (including religion).^47^

### Limitations

Limitations of our study include the perception that the number and percentages of medical students involved in these antisemitic, inflammatory, and insensitive behaviors at medical school commencements were low, therefore insignificant. However, any expression of antisemitism is deplorable; in a pluralistic society like the United States and in the heated environment of 2024, values this low can be also seen as denoting a small subset of the medical student population. The results for the overall number of medical students wearing non-official regalia and carrying protest signs may have been underestimated due to the lack of complete videos from four schools. However, it is important to recognize that having 13%–22% of medical students at some of the schools engaging in disrespectful, insensitive, and hateful behavior that is available for viewing by the public is not negligible; even a single student behaving disrespectfully or insensitively is inappropriate and can potentially create a hostile learning environment and compromise the public’s trust. Furthermore, microaggressions, particularly those directed at diverse students, are taken very seriously and often followed by consequences for violators at most US medical schools.^48^

Another limitation is that only the 25 medical schools top-rated for research (not primary care) were examined; these may not be representative of behavior in other medical schools. In the context of this study, international medical student behavior regarding antisemitism and unprofessional behavior is of interest for future research.

## CONCLUSION

Medical school faculty and leadership are role models for medical students. However, they are not always above reproach with regard to the exhibition of highly unprofessional behavior by students. Indeed, faculty may even be engaged in such egregious behaviors, for example, on social media;^8^ they may also be reluctant to remediate or discipline their students appropriately.^49^ As educators, our own activism must include reaffirming our responsibility to the public for whom we strive to provide unbiased care with clinical excellence. Education aimed at eliminating antisemitism and all forms of hate in medicine is essential. Training in respectful civil discourse, reflective practice, and sensitivity is recommended to support professionalism in all communications. Such an approach is intended to prevent hateful and insensitive displays or statements that could harm students, institutions, and their patients. United States Civil Rights Title VI cases for allegations of a hostile learning environment continue to be filed with the US Department of Education. Congress recently initiated investigations into antisemitism at UCSF and its associated medical centers under UCSF Health, as well as at Columbia University’s Vagelos College of Physicians and Surgeons in New York City.^50^ Recipients of US federal funding, such as health centers, must comply with federal laws regarding appropriate responses to such discrimination in order to avoid penalties, including the loss of federal funding.^50^ Allowing public displays of unprofessionalism may indicate an underemphasis on the core professionalism competency required by the Accreditation Council for Graduate Medical Education (ACGME) in these medical schools.^51^ Given the increasing recognition of antisemitism in US medicine,^52^ as well as increasing global antisemitism,^53^ it is prudent for medical schools and health centers to take proactive steps to address and attenuate antisemitism and all forms of discrimination to ensure a safe environment for all.

## Data Availability

Data and links to the publicly available commencement videos are available at the following link: https://uofi.box.com/s/k2tu8h9iwqyrv2vyl3lukwzaiy4qgapr

## Abbreviations

ACGME: Accreditation Council for Graduate Medical Education
BDS: Boycott, Divest, and Sanction
CI: 95% confidence interval
FERPA: United States Family Educational Rights and Privacy Act
HIPAA: United States Health Insurance Portability and Accountability Act
IHRA: International Holocaust Remembrance Alliance
UCSD: University of California at San Diego (UCSD)
UCSF: University of California at San Francisco (UCSF)
UK: United Kingdom
US: United States
